# Insight problem solving is not that special, but business is not quite 'as usual': typical versus exceptional problem-solving strategies

**DOI:** 10.1007/s00426-022-01786-5

**Published:** 2023-01-08

**Authors:** Nirit Salmon-Mordekovich, Mark Leikin

**Affiliations:** 1 Gordon College of Education, 73 Tchernichovsky St, 3570503 Haifa, Israel; 2 RANGE Center, Neuro-Cognitive Laboratory for the Investigation of Creativity, Ability and Giftedness, University of Haifa, Haifa, Israel; 3 The Edmond J. Safra Brain Research Center for the Study of Learning Disabilities, University of Haifa, Haifa, Israel

## Abstract

The intriguing phenomenon of insight (also known as the "Aha!" moment) has provoked a long-standing conflict over its cognitive mechanism. The special-process theory posits insight as a unique, unconscious mechanism. Conversely, the business-as-usual theory conceptualizes insight processing as ordinary and similar to non-insight, i.e., analytic, incremental, and attention demanding. To resolve this conflict, participants completed cognitive tests and solved four types of problems: verbal insight, spatial insight, verbal non-insight, and spatial non-insight. These problems were solved under three conditions: silence (control), inner speech suppression (articulatory suppression), and non-verbal attentional demands (spatial tapping). Interestingly, insight problem solving differed from verbal non-insight, but resembled spatial non-insight problem solving. Solving insight and spatial non-insight problems substantially benefitted from spatial and near verbal analogical thinking and convergent thinking, and little from divergent thinking. Both were unaffected by secondary tasks. Analogical thinking was associated more strongly with the generation of new solution procedures than with the retrieval of known procedures from memory, as in verbal non-insight problem solving. Analogical and convergent thinking seem to be key skills for the creation of new solutions, whether or not they are insight based. The results indicate a typical, analytic solution method consistent with the business-as-usual theory. Yet, there is also evidence for an exceptional solving method that includes rare attributes of the insightful process delineated by the special-process theory. Thus, we endorse an unequally integrated assertion that each theory reflects a different mode of thinking, the common versus the uncommon, by which insightful solutions can be produced.

## Introduction

Insight, also known as the "Eureka!" or "Aha!" moment, refers to the sudden revelation of how to solve a problem (Weisberg, 2015). Insight problems (IPs) typically present unclear information that may lead to inappropriate solutions or to no solution at all. The solver feels that the problem is unsolvable. To overcome this impasse, the problem representation should be restructured to enable reinterpretations and new directions of search for a solution (Ash & Wiley, 2006). The sudden realization of a path to a quick solution generates a surprise effect accompanied by complete confidence that the solution is indeed correct (Webb et al., 2016).

Conversely, non-insight problems (NIPs), also referred to as standard, routine, incremental, or analytic problems, contain sufficient information to allow one to clearly specify a goal and a plan, and to progress incrementally towards one absolute correct solution (Wieth & Burns, 2006). The steps towards the goal are clear and systematically evaluated, so the solver feels confident about their progress, but less confident about the final solution (Danek et al., 2014).

"Insight" and "non-insight" denote not only types of problems, but also types of solution strategies (Kounios et al., 2008). The insight strategy involves automatic restructuring of the initial problem representation outside of awareness and only the solution surges suddenly into consciousness. Thus, the means of solution do not rely on inner speech and are ineffable (Ball et al., 2015). Conversely, the analytic non-insight strategy involves a conscious, step-by-step search for a solution. It relies on reasoning and working memory (WM), which maintains the active record of the solving plan (Chuderski & Jastrzębski, 2018), and on inner speech, which supports retention of the sequential information (Baddeley, 2000).

Both strategies can result in successful IP solving (Salmon-Mordekovich & Leikin, 2022; Weisberg, 2015). Tasks that are traditionally categorized as "insight" can be solved without an accompanying "Aha!" experience (Danek et al., 2016). Therefore, the traditional classification of tasks into insight and non-insight is insufficient. In the current study, defining IPs and NIPs was based on both forms of classification: task and strategy, as indicated by self-reports of solvers' solution experience. Problems that were analyzed as "insight" met these two conditions; not only were they previously labeled as pure or classic in the literature, but participants reported having solved them by insight.

The phenomenon of insight has intrigued theorists and researchers for at least a century. It lies at the heart of a long-standing conflict between the early “special-process” theory that emphasizes insightful processing as unique, associative, unconscious thus, non-reportable (Ash & Wiley, 2006; Ball et al., 2015; Danek et al., 2014), and the “business-as-usual” theory that claims that similar incremental, controlled cognitive processes are involved in both IP and NIP solving (Chuderski & Jastrzębski, 2018; Fleck & Weisberg, 2013; Gilhooly et al., 2010). This conflict is still relevant for discussion as neither theory can fully account for IP solving.

In an attempt to resolve this theoretical conflict, studies often compare the role of individual cognitive functions in IP solving with their critical role in NIP solving. Such studies assume that differences and/or contributions of associative and divergent thinking, and of inner speech suppression support the special-process theory (e.g., DeYoung et al., 2008), whereas similarities and/or contributions of inner speech, WM, reasoning, convergent, and analytic skills support the business-as-usual (e.g., Chuderski & Jastrzębski, 2018). These assumptions are supposedly easy to test but, as the review below shows, the findings of these comparative studies are controversial.

### Individual cognitive differences underpinning problem solving

#### Divergent thinking and convergent thinking

Since Guilford (1958) first differentiated between divergent and convergent thinking and designated the former as critical to creativity, these abilities have been discussed in the field of problem solving. Divergent thinking refers to the ability to generate a variety of answers, some unconventional, whereas convergent thinking entails arriving at a single correct solution.

Although divergent thinking was assumed to be unique to insight (DeYoung et al., 2008), some evidence showed that it is associated with both IP and NIP solving (Gilhooly & Murphy, 2005). Moreover, recently, convergent thinking was found to be a stronger predictor of successful IP solving than divergent thinking (Webb et al., 2017). Convergent thinking may support the assessment of the appropriateness and quality of ideas generated through divergent thinking, creating logical connections between them and enabling incremental progress toward the best solution (Lee & Therriault, 2013). This study contrasts the relationship of divergent thinking and IP with NIP solving. To date, research in this field has been scant and results often conflicting.

#### Analogical thinking

The notion that reasoning by analogy may underlie insight is contentious. According to the business-as-usual view, both IP and NIP solving begin by searching the memory for a compatible analogous problem similar in structure or principle. If found, the solution might be retrieved from the memory and applied to the current problem. If the solution fails, then the new information obtained from the failure prompts a new analysis of the problem (i.e., restructuring) (Weisberg, 2015). Conversely, according to the special-process theory, analogical thinking produces solutions based on existing information; therefore, it is probably ineffective in generating novel insightful solutions.

Most studies on analogical thinking and insight have explored historic anecdotes of insightful discoveries or experiments of primed contexts (e.g., Gick & Holyoak, 1980). In these experiments, first, anecdotal information about a problem and its solution is presented; only then is it followed by the current problem. To solve the problem, the initial information needs to be applied analogously. Apparently, with no salient, superficial similarity between problems, noticing the connection between them is difficult. Retrieval and transfer of analogous elements are neither automatic nor spontaneous (George & Wiley, 2018). Thus, the contribution of analogical thinking to IP solving remains ambiguous. This study examines the relationship between IP solving and analogical thinking, however, as a person's individual ability since few studies have tested this ability for purposes other than assessing its potential role in insight.

#### Inductive reasoning

Analytic problem solving typically relies on systematic reasoning of complex data based on the solvers' experiences and learning. Solvers are often required to generalize their observations into a hypothetic principle then, integrate it within the solving process, and finally, validate the principle as suitable. A typical measure of problem solving through abstract reasoning is Raven’s advanced progressive matrices (RAPM) (Raven et al., 1996). Findings of testing the RAPM alongside IPs have been contradictory as to whether abstract reasoning contributes to IP solving (Fleck, 2008) or not (Gilhooly & Murphy, 2005).

### Inner speech and problem solving

Inner speech was modeled as a subcomponent of WM (Baddeley, 2000). This model includes the phonological loop and the visuospatial sketchpad, which temporarily store auditory–verbal and visuospatial representations, respectively. Content represented in the phonological loop decays rapidly. To revive it, it must be rearticulated by inner speech. Inner speech supports multi-step task planning and maintains incomplete plans in WM, while they are being assessed and revised (Lidstone et al., 2010).

The role of inner speech in IP solving is controversial. Schooler et al. (1993) provided influential evidence supporting the special-process view by demonstrating that verbalizing thoughts aloud while trying to solve problems impaired performance on IPs, but did not affect NIPs. Macchi and Bagassi (2012) further clarified that it is the imposition of an explicit stepwise process, rather than language itself, that hinders insight. Ball et al. (2015) reinforced these conclusions by demonstrating facilitation of insightful solutions when opportunities for internalized speech-based processing were reduced, thereby enabling more effective unconscious, non-reportable processes.

Gilhooly et al. (2010), however, disagreed, and noted that the majority of IPs in the Schooler et al. study were spatial, while the NIPs were mostly verbal. The researchers claimed that verbalization is presumed to impair performance on spatial problems since it imposes inefficient verbal coding rather than appropriate spatial coding. This argument might also counter the later studies mentioned above, which applied spatial IPs only. For this reason, Gilhooly et al. examined spatial and verbal IPs and NIPs separately, showing a greater verbalization effect on performance of spatial versus verbal problems regardless of whether or not they were insight based. This evidence supports the business-as-usual theory, which argues that restructuring occurs through incremental reportable steps.

In summary, studies that present contributions of inner speech, reasoning, convergent and analogical thinking to IP solving support the business-as-usual theory, whereas contributions of divergent thinking and the suppression of inner speech support the special-process theory. The goal of this study is to provide an integrative account that reconciles these theories. Based on this literature review, we proposed that each theory reflects a different solution strategy, both of which can produce successful solutions. The business-as-usual theory indicates the analytic strategy, which is the typical problem-solving method, whereas the special-process theory presents the insight strategy, which is exceptional. We assumed that IPs are solved routinely through analytic strategies as are NIPs. Seldom is the insight strategy used. Furthermore, the combination of the two theories at the process level, that is, the combination of insight and analytic strategies or divergent and convergent thinking, could reflect these uncommon cases. Divergent thinking produces diverse ideas, but it is insufficient on its own. Insight is probably not a product of fluent retrieval of ideas or free associations. It is the convergence of these ideas based on a new concept that is critical and would ultimately form an insightful solution.

Accordingly, we investigated the controversial involvement of inner speech, convergent and divergent thinking in IP solving, as well as the role of analogical thinking, which has been under-addressed.

We tested the following hypotheses:

1. The business-as-usual theory advocates the common, routine, analytic strategy. Thus, we anticipate similar connections between the tasks of inductive reasoning and analogical thinking with both IP and NIP solving, and we expect convergent thinking to be more substantial than divergent thinking.
2. The special-process theory presents the uncommon, special strategy of solving IPs. We therefore expect the core milestones of the theory, impasse and insight, to be rare and the contribution of divergent thinking to be negligible although statistically significant.
3. Precluding inner speech and distracting attention should not interfere with IP solving since it requires the generation of new, non-linear procedures. Conversely, NIPs are solved through retrieval of sequential, pre-prepared, planned procedures. Therefore, NIP solving would benefit from enabling inner speech and optimal attention since they support storing and following sub-goals and interim solutions in WM.

## Methods

### Participants

We recruited 115 undergraduate students (92 women, *M*_age_ = 26.25) as paid volunteers. This sample size was calculated using G*Power software based on RM MANOVA (medium effect size *f* = 0.25, *α* = 0.01, power = 0.95). Sample size based on a one-tailed correlation (medium effect size *ρ* = 0.3, α = 0.01, power = 0.80) was estimated to be 107. Sample size based on a four-step regression and five predictors (medium effect size *f*^*2*^ = 0.15, α = 0.05, power = 0.80) was estimated to be 85. Participants were native Hebrew-speakers, who reported no prior diagnosis of language and learning disabilities, attention deficit disorders, or chronic hearing impairments. All participants gave their written informed consent.

### Materials

#### Problems

Other researchers previously used the problems, and labeled the IPs as pure or classic and the NIPs as analytic or incremental. Problems were attempted to match with an approximately 50% solution rate. However, since solution rates of most problems were unspecified or inconclusive across studies, problems from online sources were added after being evaluated by a preliminary pilot study. Appendix A presents sample problems.

**Verbal insight problems.** “Car”, “Checkers”, “Ladder” (DeYoung et al., 2008), “Prisoner” (Schooler et al., 1993), “Lake” and “Blind” (Gilhooly et al., 2010).

**Spatial insight problems.** “Triangle of Coins” (solved concretely), “Pigpen” (Schooler et al., 1993), “Farm” (Gilhooly et al., 2010), “Four Dots” (Chuderski & Jastrzębski, 2018), “Bus” and “Matchsticks” (online sources).

**Verbal non-insight problems*.*** “Bachelors”, “Committee”, “Flowers” (Wieth & Burns, 2006), “Schedule” (Gilhooly et al., 2010), “Sisters” and “Ski” (online sources).

**Spatial non-insight problems. “**Four Coins” (Schooler et al., 1993), “Tower of Hanoi” (5-disc, solved concretely) (Fleck, 2008), “Trace” (Webb et al., 2017), “Wolf, Sheep and Cabbage” (Gilhooly et al., 2010), “Squares” and “Cubes” (online sources).

#### Individual difference measures

**Divergent thinking test. *The alternate uses task*** (AUT, Guilford, 1967). Participants generated as many unusual uses as possible for two everyday items in three minutes each. The total number of suggestions given determined the fluency score. The flexibility score was the number of different categories used. Originality was scored by giving one point for responses by 3–10% of the respondents, two points for responses by less than 3%, and three points to unique responses (DeYoung et al., 2008).

**Convergent thinking test. *Remote associates test*** (RAT, Nevo & Levin, 1978) comprises 25 items. Each item displays three unrelated words (e.g., lie, flag, egg). The task is to retrieve a single word association that relates to each word in the triad (e.g., white).

**Inductive thinking test. *Raven’s advanced progressive matrices*** (RAPM, Raven et al., 1996) (shortened version, Salmon-Mordekovich & Leikin, 2022) comprises a series of abstract figures arranged in a 3 × 3 matrix in which one figure is missing. The task is to discover rules by which to identify which one of eight alternatives completes the matrix. RAPM has also been used as a non-verbal estimate of convergent thinking (Akbari Chermahini et al., 2012; Webb et al., 2017).

**Analogical thinking tests. *Verbal analogy task*** includes 18 items adapted from admission exams for universities. Each item consists of two-word pairs sharing a common relationship. The fourth word is missing and should be identified from among five options. Analogies are of two types: semantically far, in which both pairs are of different domains (e.g., juice: beverage–coin: money), and semantically near, in which both pairs share the same domain (e.g., juice: beverage–marshmallow: candy).***Visuospatial Analogy task*.** Eighteen visuospatial items, collected from psycho-technical screening tests, are displayed in the form of A:B–C:D, with element D missing. The task is to complete the missing object with one of four options so that both pairs share an analogous visuospatial relationship.

All of the tests except the AUT were scored by the percentage of correct solutions.

### Procedure

**Fig. 1:**
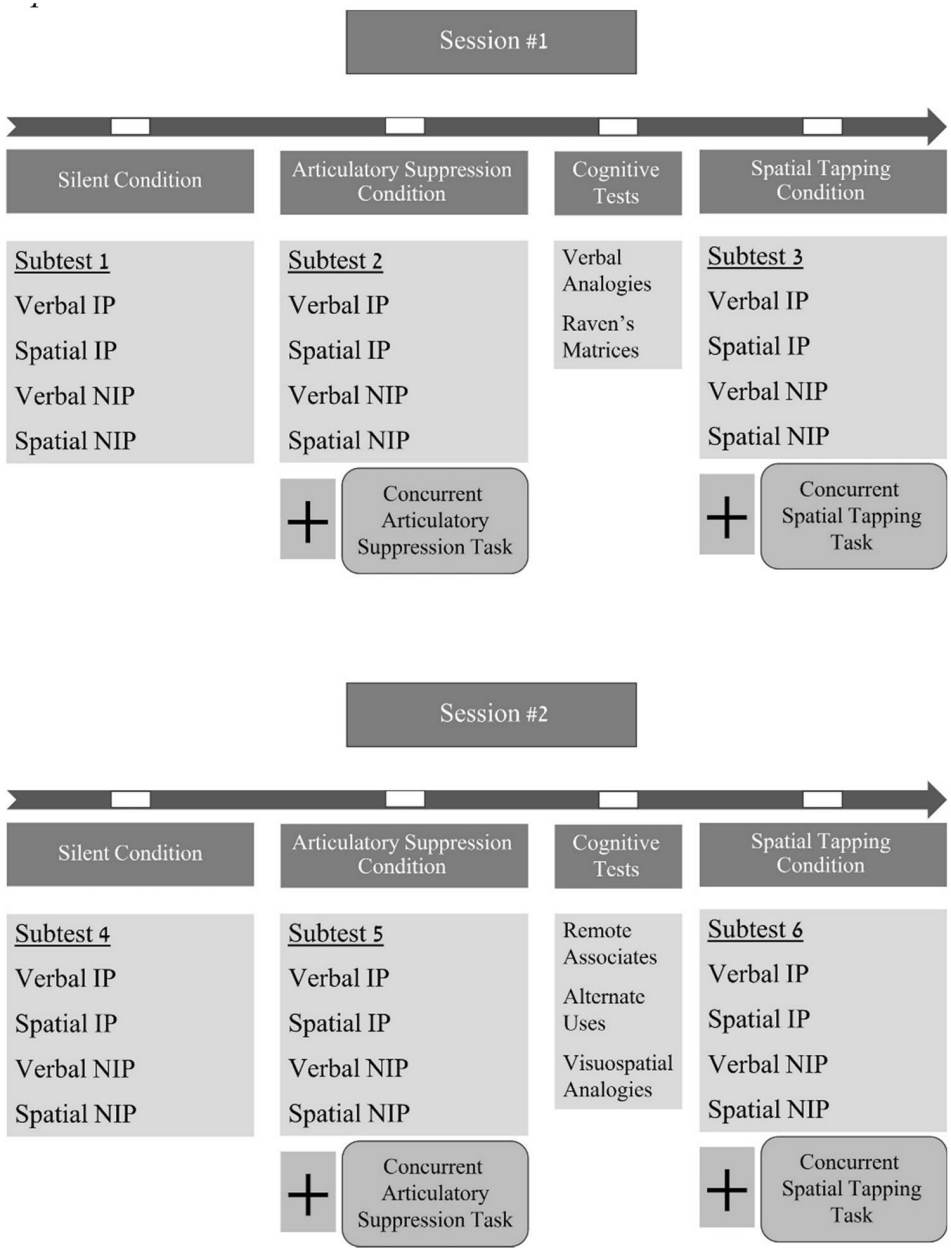
Experiment's structure. Note: Order of conditions and problems were randomized within and across subtests. *IP* insight problems, *NIP* non-insight problems

Participants were randomly assigned to two sessions, held within a week. Each session included three subtests of problems to be solved with paper and pencil under three randomized conditions, and in-between computerized cognitive tests (Fig. 1). Problems of four types—verbal IP, spatial IP, verbal NIP, and spatial NIP—were randomized so that every subtest included one of each. One practice problem of each type preceded these subtests. Each problem was allotted up to four minutes. Problems were solved silently (control), while counting from 2001 to 2005 repeatedly aloud (articulatory suppression), and while repeatedly tapping the numbers 1, 2, 3, 6, 5, 4 on a keyboard, forming a rectangular pattern (spatial tapping). Participants tapped with their non-dominant hand hidden by a box. They were encouraged to count and tap at a constant rate and were monitored by the researcher. The purpose of the second condition was to suppress the phonological loop (Baddeley, 2000), thereby precluding inner speech. The third condition was to suppress the visuospatial sketchpad (Robbins et al., 1996), thereby imposing comparable attentional demands, but not affecting inner speech.

Prior to the experiment, participants were briefed on how to distinguish IP from NIP solving and on impasses they might encounter. They were instructed to report impasses by clicking a button and to indicate after each problem retrospectively whether they experienced an insight (instructions as in Webb et al., 2016). Finally, participants were asked if they were familiar with the problem and if so, we removed it from the analyses.

### Analysis

IPs were classified not only by the task, but also by its solving process. The classic IPs were analyzed as IPs provided the subject reported experiencing an insight. A composite solution score for IP solving (IP score) was computed for each of the participants based on their averaged success in solving verbal and spatial IPs in each of the three experimental conditions. Similarly, a composite score for NIP solving (NIP score) was computed for each solver. We removed problems that yielded solution rates greater than 85% (Schedule) or lower than 15% (Farm, Ski) from the analyses. In addition, the Triangle problem strikingly presented an exceptional 39% decline in success rates in the spatial tapping condition vs. the silent condition. This problem was the only IP that used accessories. Participants were required to form a triangle from coins and move them with one hand while tapping a rectangular pattern with the other hand. It is likely that these two concurrent motor tasks presented a significant obstacle to coordination substantially impairing participants’ ability to solve this problem. Therefore, we eliminated its performance from the analyses under this condition.

Pearson correlation coefficients were computed to assess the relationships between IP and NIP scores and each of the individual cognitive tests. The significance level was set at 0.01. Since effect sizes in social sciences are often small, to represent a practical significant effect size, Pearson's *r* is recommended to be anchored to a minimum of 0.20 (Ferguson, 2009). In the context of the research questions, significant (*p* < 0.01) correlation coefficients larger than 0.25 were assumed to indicate significant relationships between variables and values over 0.40 to indicate moderate relationships. Fisher's *Z*-tests were performed to test the significance of the differences between correlation coefficients of the cognitive measures with IP vs. NIP scores.

Additionally, we conducted hierarchical multiple regression analyses to examine whether the same cognitive skills would predict IP and NIP solving. IP and NIP solving were separately regressed on the cognitive variables including a compound score of divergent thinking, which was computed as the average of the *Z*-scores of fluency, flexibility, and originality.

In case the two theories reflect contradictory discrete models of IP solving, then, according to the special-process theory, out of all the individual difference measures, the AUT indices (i.e., measures of divergent thinking) would present the highest Pearson correlation coefficients, and substantially account for the largest portion of the variance in IP solving, however would be insignificant factors in NIP solving. Fisher's *Z*-tests would confirm these differences. Conversely, the business-as-usual theory would predict that all of the individual difference measures, except for the AUT indices, would present moderate correlations with both IP and NIP solving, and would explain a substantial portion of the variance in both types of solving.

Nevertheless, we endorse an unequally combined account in which both IPs and NIPs are routinely solved via common analytic strategies, as the business-as-usual theory posits, and rarely by insight strategy. That is, the unique insight strategy, delineated by the special-process theory, is special in terms of how uncommon it is, but it is not that special since it is not unique to IP solving and could also be applied in successful NIP solving. Thus, we expected both the IP and NIP performances to similarly show significant, positive, moderate correlations with the test scores of RAPM, RAT, and analogies. These variables should substantially account for the variance of both IP and NIP solving. Moreover, AUT indices should also present significant relationships with both IP and NIP solving; however, it is expected to account for a very small portion of their variance.

We also computed performance scores for verbal IPs, spatial IPs, verbal NIPs, and spatial NIPs to more fully examine the similarities and differences between them. Each score is the average of the scores achieved in each of the experimental conditions. Pearson correlation analyses were conducted to examine the relationships between these performance scores and the cognitive tests. Path analysis was used to reveal predictive cognitive measures of successful problem solving.

Finally, we conducted a Friedman test and a post hoc analysis with Wilcoxon signed-rank test with a Bonferroni correction for each problem type to compare the effects of the three experimental conditions on performance.

## Results

### Individual cognitive differences

Table 1 presents correlations between the individual cognitive measures and performance on IPs and NIPs. All tasks except for the far verbal analogy were significantly correlated with both IP and NIP solving. Fisher's *Z*-tests were performed to examine differences between correlations of the cognitive tests with IP vs. NIP scores. Results showed stronger correlations for spatial (*p* = 0.01) and near verbal (*p* = 0.04) analogical thinking with IP than with NIP scores.

**Table 1:** Descriptive statistics and correlations between individual cognitive measures and mean scores of insight and non-insight problem solving

	M	SD	Insight problems	Non-insight problems
Insight problems	0.52	0.23	1	0.44**
Non-insight problems	0.48	0.23	0.44**	1
RAPM	69.77	12.59	0.45**	0.42**
RAT	30.74	13.05	0.37**	0.41**
Far analogy	70.22	15.23	0.05	– 0.02
Near analogy	75.03	18.03	0.43**	0.27**
Spatial analogy	72.66	14.45	0.55**	0.36**
AUT fluency	7.03	3.06	0.32**	0.28**
AUT flexibility	4.63	1.7	0.40**	0.31**
AUT originality	11.82	6.27	0.30**	0.29**

*RAPM* Raven’s advanced progressive matrices, *RAT* remote associates test, *AUT* alternate uses task

**p < 0.01

We conducted two hierarchical multiple regression analyses: one on the IP score and the other on the NIP score as the dependent variables. The cognitive measures were the independent variables in both analyses (Table 2). Since both types of problem solving converge in a closed-ended solution, RAT being a measure of convergent thinking was included in the first block. RAPM, which is also convergent in nature, captures inductive reasoning typical of analytic solving, and it was entered in the second block. Analogical thinking, typically attributed to analytic solving, was entered in the third block. Divergent thinking, typically attributed to insight solving, was entered last. As shown in Table 2, RAT and RAPM significantly explained 26% of the variance in IP solving. Analogical thinking explained an incremental 21% of the variance above the variance accounted for by the measures of convergent and inductive thinking. Adding divergent thinking to the regression model accounted for an additional 2% of the variance.

**Table 2:** Summary of hierarchical regression analyses for cognitive variables predicting insight and non-insight problem solving

Block	Variable	Insight problems				Non-insight problems			
		β	*t*	*F* change	*R*^2^ change	β	*t*	*F* change	*R*^2^ change
1				17.32***	0.13			23.14***	0.17
	RAT	0.37	4.16***			0.41	4.81***		
2				18.73***	0.13			15.26***	0.10
	RAT	0.26	3.01**			0.32	3.73***		
	RAPM	0.37	4.33***			0.33	3.91***		
3				20.63***	0.21			3.68*	0.05
	RAT	0.18	2.41*			0.28	3.31**		
	RAPM	0.20	2.39*			0.26	2.79**		
	Near Analogy	0.29	3.91***			0.17	2.21*		
	Spatial Analogy	0.34	4.14***			0.14	1.50		
4				4.33*	0.02			2.68*	0.02
	RAT	0.15	2.04*			0.25	2.99**		
	RAPM	0.18	2.27*			0.25	2.68**		
	Near Analogy	0.27	3.65***			0.14	1.66		
	Spatial Analogy	0.33	3.99***			0.13	1.35		
	AUT	0.15	2.08*			0.14	1.64		

*RAT* remote associates test, *RAPM* Raven’s advanced progressive matrices, *AUT* alternate uses task

*p < 0.05, **p < 0.01, ***p < 0.001

The regression analysis of NIP solving showed that RAT and RAPM explained 27% of its variance. Analogical thinking explained an additional 5% of the variance. Divergent thinking accounted for an additional 2%. However, its contribution was insignificant. Accordingly, individuals with better convergent, inductive, and analogical skills are more likely to succeed in solving both IPs and NIPs.

Results of the regression and correlational analyses support the business-as-usual theory. Nevertheless, analogical thinking is associated more substantially with IP than with NIP solving. This finding suggests the need for a deeper examination of potential differences between subtypes of IP and NIP solving. Therefore, correlational analyses were conducted according to problem modality (Table 3). Solution scores of all subtypes—verbal and spatial IPs and NIPs—were significantly positively correlated with the convergent and inductive thinking measures. Verbal and spatial IP solving had significant, albeit weak, correlations with the fluency and originality indices of divergent thinking; flexibility was more substantial. These indices also correlated with the spatial NIP score, however not with the verbal NIP score. Both spatial and near analogical tasks were significantly correlated with both verbal and spatial IP solving as well as spatial NIP solving. However, these tasks were not associated with verbal NIP solving. Fisher's *Z*-tests confirmed the stronger associations for spatial (*p* = 0.008, *p* = 0.001, *p* = 0.003) and near verbal (*p* = 0.007, *p* = 0.003, *p* = 0.001) analogical thinking with verbal IP, spatial IP, and spatial NIP, respectively, than with verbal NIP scores.

**Table 3:** Correlations between mean scores of verbal and spatial insight and non-insight problem solving and individual cognitive measures

	Verbal IP	Spatial IP	Verbal NIP	Spatial NIP
RAPM	0.31**	0.44**	0.31**	0.37**
RAT	0.35**	0.27**	0.35**	0.33**
Near analogy	0.35**	0.36**	0.06	0.38**
Far analogy	– 0.05	0.13	– 0.01	– 0.03
Spatial analogy	0.43**	0.48**	0.16	0.44**
AUT fluency	0.26**	0.28**	0.15	0.30**
AUT flexibility	0.34**	0.32**	0.17	0.33**
AUT originality	0.24*	0.26**	0.17	0.30**

*IP* insight problems, *NIP* non-insight problems, *RAPM* Raven’s advanced progressive matrices, *RAT* remote associates test, *AUT* alternate uses task

*p < 0.05, **p < 0.01

**Fig. 2:**
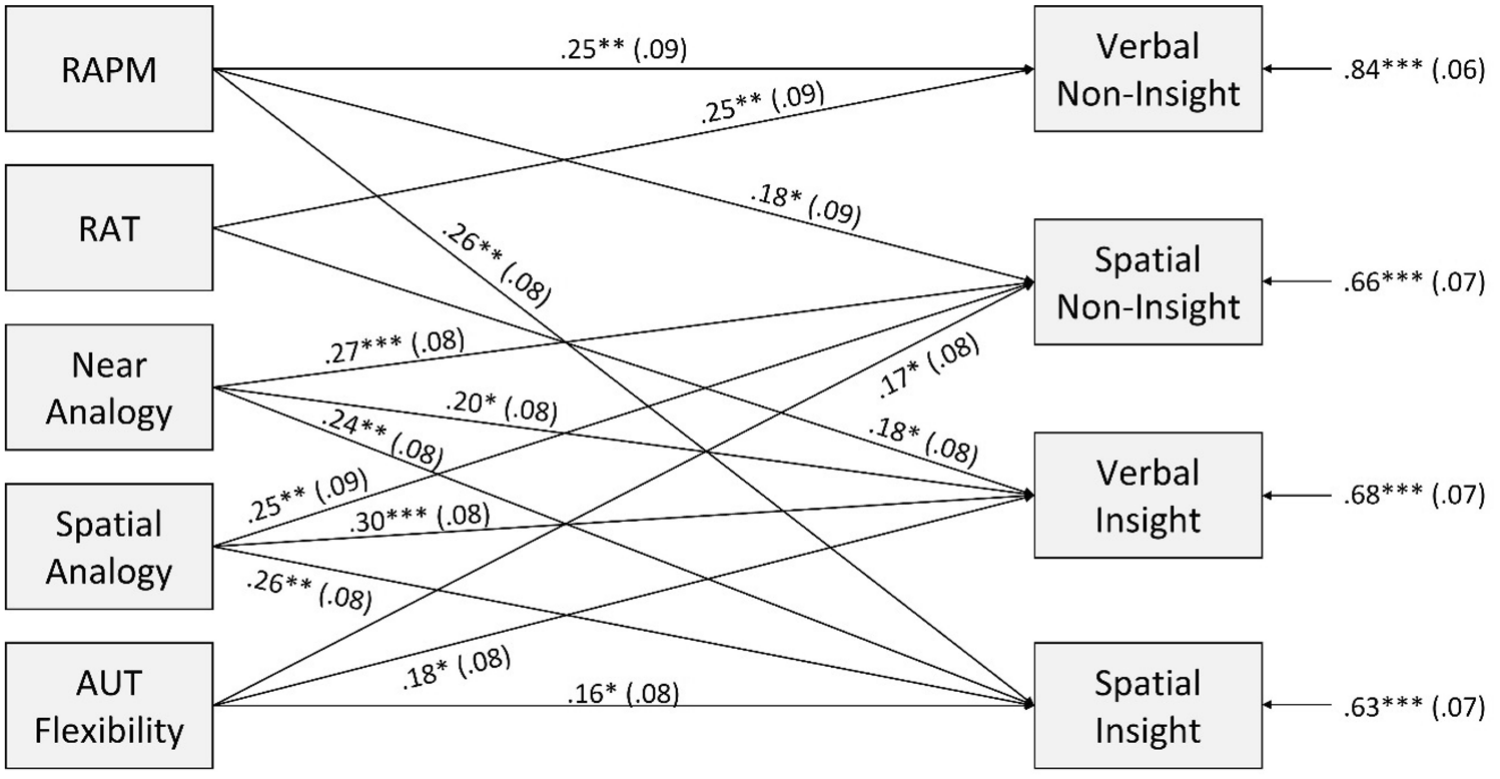
Cognitive predictors of verbal and spatial insight and non-insight problem solving. *RAPM* Raven’s advanced progressive matrices, *RAT* remote associates test, *AUT* alternate uses task. *p < 0.05, **p < 0.01, ***p < 0.001

Analogical and divergent thinking differentiated IP and spatial NIP from verbal NIP solving. Seemingly, IP solving was similar to spatial NIP solving but different from verbal NIP solving. Based on these unexpected results, we hypothesized that solving verbal IPs, spatial IPs, and spatial NIPs benefits from analogical thinking and flexible thinking. However, these cognitive measures are insignificant for verbal NIP solving since they are not correlated. Out of all the cognitive variables tested, verbal NIP solving should rely only on convergent and inductive thinking. We conducted path analysis to examine these hypothesized relationships simultaneously. Figure 2 presents the resulting standardized regression weights of the significant paths for each type of problem solving. This path model fits the data well (*χ*^2^_(6)_ = 3.65, *p* = 0.72; CFI = 1.00, TLI = 1.07, RMSEA < 0.001, SRMR = 0.02).

IP and spatial NIP solving were predicted by flexibility, verbal and spatial analogical thinking, and convergent thinking with the last being verbal (RAT) for verbal IP and spatial (RAPM) for spatial IP and NIP. These variables explained 32, 37, and 34% of the variance in verbal IP, spatial IP, and spatial NIP solving, respectively.

### Insight and impasse

Inspection of the successfully solved IPs showed that 88.5% elicited insight as reported by the participants. However, only 7.97% of the problems reported as eliciting insight included a state of an impasse.

Two participants (1.7%) reported an impasse while silently solving verbal NIPs whereas 37, 41, and 21% of participants reported impasses while silently solving verbal IPs, spatial IPs, and spatial NIPs, respectively.

### Effects of experimental conditions

**Fig. 3:**
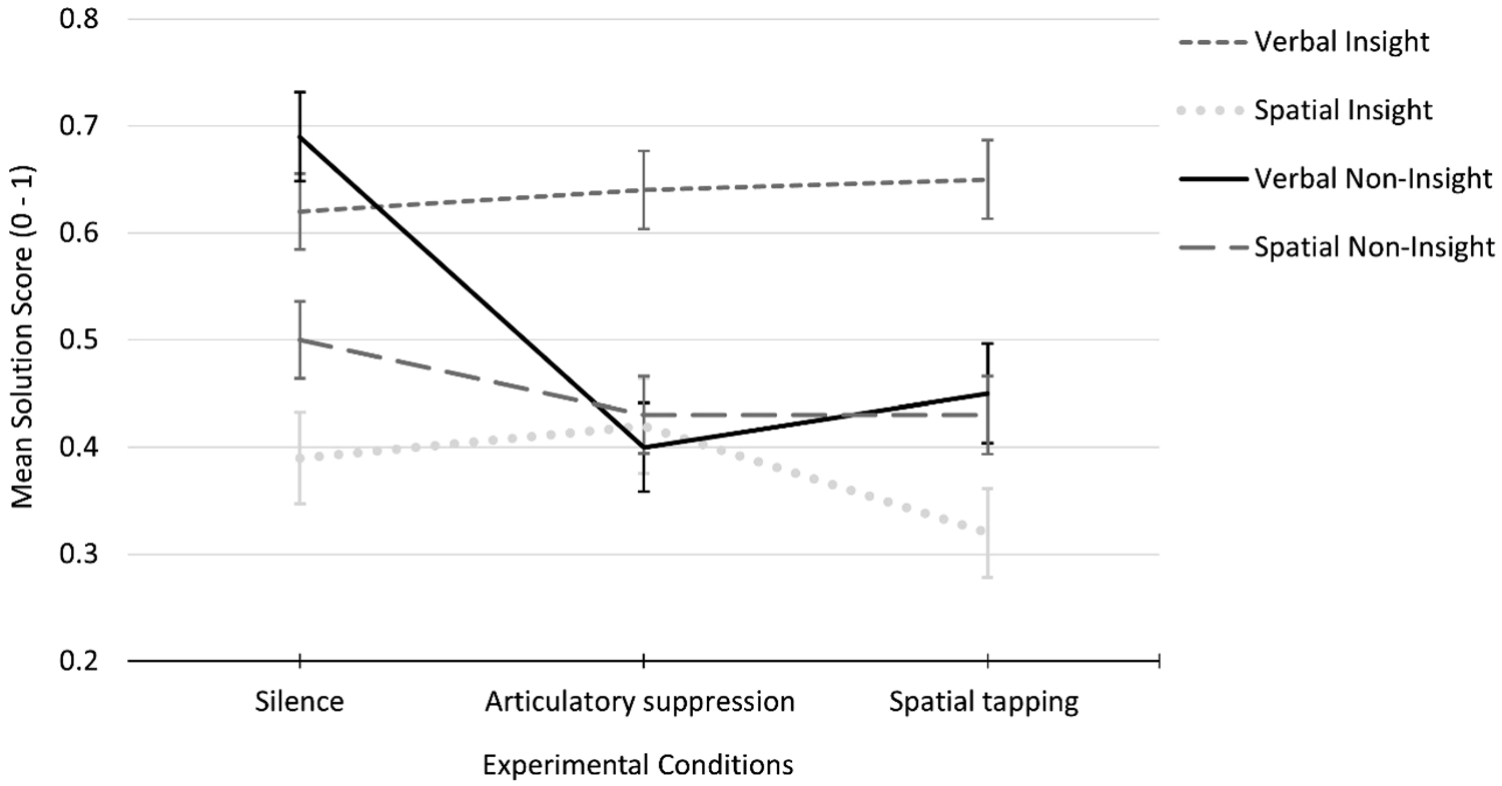
Mean solution scores of verbal and spatial insight and non-insight problems across experimental conditions. Error bars represent the standard errors of the mean

The Friedman test results on problem-solving accuracy across the experimental conditions are shown in Table 4. Verbal NIP scores differed significantly across the experimental conditions, as expected. Post hoc analysis with the Wilcoxon signed-rank test showed that solving these problems while repeatedly reciting (*p* < 0.001) or repeatedly typing a series of numbers (*p* < 0.001) was significantly different than solving problems silently. The performance was significantly reduced by 29% in the articulatory suppression condition and 24% in the spatial tapping condition. There was no significant difference between the two secondary-task conditions (*p* > 0.05). Thus, solving verbal NIPs seems to be equally vulnerable when performed concurrently with either a verbal or a motor task. Conversely, solution rates of verbal IPs, spatial IPs, and spatial NIPs did not significantly differ across the experimental conditions. Figure 3 summarizes the results of solution scores of the four types of problems across the three conditions.

**Table 4:** Friedman test statistics of problem-solving accuracy as a function of experimental conditions

Condition	Verbal IP			Spatial IP			Verbal NIP			Spatial NIP		
	Med	M	SD	Med	M	SD	Med	M	SD	Med	M	SD
Silence	0.5	0.62	0.38	0	0.39	0.48	1	0.69	0.43	0.5	0.5	0.38
Articulatory suppression	0.5	0.64	0.39	0	0.42	0.47	0.5	0.4	0.44	0.5	0.43	0.39
Spatial tapping	0.5	0.65	0.39	0	0.32	0.4	0.5	0.45	0.47	0.5	0.43	0.38
*χ*^2^_(2)_		0.02			1.74			22.53***			2.57	

*IP* insight problems, *NIP* non-insight problems

***p < 0.001

In summary, considering that 92% of the IPs successfully solved did not include a state of impasse, and given the resemblance of IP to spatial NIP processing shown by the path analysis, the correlational findings, and the dual-task analysis, insight processing is not unique and exhibits analytic processing as posited by the business-as-usual theory. Still, we cannot overlook that both types of IPs differed from verbal NIP solving, which could corroborate the special-process theory and illustrate the conflicting evidence in the literature.

## Discussion

The aim of this study was to provide an integrative account of IP solving that would advance a resolution of the conflict between the special-process and the business-as-usual theories. This study presents novel findings: IP solving was consistently similar to spatial NIP solving, but consistently different from verbal NIP solving. Spatial and near verbal analogical thinking, flexibility (divergent thinking), and the effects of articulatory suppression and non-verbal attentional load differentiated IP from verbal NIP solving. However, the mechanism of IP solving is not unique since it shared these cognitive processes and conditions' effects with spatial NIP solving. Accordingly, while insight processing is not that ''special'', business is not totally ''as usual''.

### Analogical thinking

Results showed that spatial and near verbal analogical thinking were significant predictors of IP solving, explaining one-fifth of its variance. Participants who excelled in the near analogical thinking task also excelled in solving both types of IPs. This finding confirms that analogical thinking does lead to innovative solutions, not only to existing ones.

Far verbal analogy thinking was not associated with problem solving. The near and far analogy tasks seem to capture different aspects of thinking. Near analogies share surface features of a similar knowledge domain so they are easier to detect and access (Holyoak & Koh, 1987). Inference and transfer of relations between pairs are faster and nearly effortless; hence, the near-analogy strategy may account for the quick-solving nature of IPs. Far analogies, on the other hand, contain a pair whose relationship is more abstract, and mapping it onto the second pair in a different domain is more difficult and less obvious. Far analogical thinking may have been expected to correlate with IP solving since both challenge the solver requiring an extreme change of perspective. However, without a prompt to connect remote analogies that differ in their surface features, making such connections is unlikely (George & Wiley, 2018). Our findings are compatible with Dunbar's (1995) conclusion that near rather than far analogies should be employed as a problem-solving strategy, as they lead to a conceptual change more efficiently. Successful solvers possess a richer semantic domain; they discern more similarities within it, thus making more productive analogies. Comparing the analogous problems strengthens their common features and correspondent differences (Gentner et al., 2003), consequently reinforcing the focus on certain aspects of the problem or making new assumptions about others, which leads to problem restructuring.

Interestingly, results showed that spatial analogical thinking also predicted success in solving IPs. Hence, solving IPs seems to benefit from efficient retrieval of both verbal and visuospatial representations from long-term memory and relies heavily on reasoning. Since spatial IPs were presented with diagrams, one may expect a spontaneous triggering of visuospatial analogical thinking (Casakin & Goldschmidt, 2000). However, given that solving verbal IPs also significantly benefited from spatial analogical thinking implies the possibility that non-verbal reasoning supports the insightful process in general, and that certain aspects of insight are abstract and non-linguistic.

Analogical thinking is typical of the solution search stage in which the solver first attempts to match the problem with prior knowledge and experience in long-term memory (Weisberg, 2015). Since the insightful solution should be innovative, the revelation that analogical thinking is such a strong contributor to IP solving may seem surprising. We propose that this significant contribution extends beyond the initial search stage. Analogical thinking is liable to highlight elements of the problem that could be linked in an exceptional way to new ideas raised at a later stage of restructuring the representation of the problem (i.e., convergent thinking). Thus, the insightful solution may be a product of this remote association. The combination and reorganization of knowledge structures into new knowledge rely substantially on analogical reasoning (Mumford & Martin, 2020). Indeed, in our study, convergent and analogical thinking explained nearly half of the variance in IP solving. Our proposal corresponds with the business-as-usual notion that analytic processing may underlie insightful solutions, including the stage of restructuring (Fleck & Weisberg, 2013; Weisberg, 2015), but this idea requires further testing.

### Divergent and convergent thinking

Consistent with previous research, this study found that flexibility is a significant predictor of insight (DeYoung et al., 2008), while fluency and originality are associated with IP solving, but they lack predictive power. We conclude that insight is not necessarily a product of generating original ideas or as many ideas as possible; rather, insight relies more significantly on the ability to generate distant ideas across different categories.

Although divergent thinking is typically attributed to IP solving (DeYoung et al., 2008), in our study, it explained only 2% of the variance. Thus, applying divergent thinking in IP solving seems atypical. Flexible thinking is insufficient on its own, and convergent thinking seems to contribute more substantially to insight processing. More precisely, insight appears to be a product of a convergent association rather than of free associations that are flexibly generated.

If divergent thinking is utilized to retrieve distant ideas, probably the convergence of a few of these ideas into a completely different concept, ultimately forming the insightful solution, might be more critical. The more diverse these ideas are, the more likely that an unusual connection between them will be revealed, thus arriving at an unconventional solution. In other words, flexible (divergent) thinking seemingly activates remote elements, whereas convergent thinking connects them via a novel insightful association. Creativity studies substantiate this; retrieving associations from distant clusters, and selecting and recombining semantic knowledge increase the probability of generating creative solutions (Benedek et al., 2012; Mednick, 1962).

### Inner speech and attention

Participants in this study solved problems under three conditions: silence (control), suppression of inner speech (articulatory suppression), and non-verbal attentional load (spatial tapping). Comparing the effects of these conditions on success rates distinguished verbal NIP from IP and spatial NIP solving, as did the correlational analyses and the path model. Only verbal NIP solving was significantly impaired by the articulatory suppression and spatial tapping tasks compared to the controls. Inner speech aids in planning solutions (Lidstone et al., 2010). When suppressed, success in solving multi-step NIPs is expected to diminish. These results demonstrate how vulnerable the processes involved in solving verbal NIPs are to attentional overload in general. Having fewer cognitive resources available significantly disrupted performance.

In contrast, performance on IPs was not significantly affected by secondary tasks. Neither suppression of the phonological loop nor the visuospatial sketchpad disrupted their performance, indicating that these two components of WM, although critical to verbal NIP solving, are not significantly involved in insight processing. This result is compatible with our findings that insightful solvers seem to rely on analogical thinking and, to a lesser extent, on flexible thinking. They retrieve analogies and ideas in a multi-directional manner rather than retrieving a linear planned procedure, as in the case of verbal non-insight processing. Thus, ongoing plans include less information to retain, to assess, or to revise in WM. Moreover, only near analogical thinking predicted successful IP solving, whereas far analogical thinking was unrelated. Since associations that characterize the near analogies are stronger, the analogous process should be rapid, effortless, and not place a heavy load on WM.

Insight processing appears to rely less on resource-demanding and linguistic strategies. It endures interference in verbal or spatial encoding regardless of whether the problems are verbal or spatial. Concurrent spatial tasks did not impair solving spatial IPs, nor did a concurrent verbal task impair solving verbal IPs. The mechanism of insight appears to be modality-general and of higher-order cognitive functioning. It involves an abstraction of input and its integration in memory. This argument is supported by our previously described results that both verbal and spatial IP solving rely on non-verbal analogical reasoning, thereby reinforcing evidence that insight, at least in part, is not speech-based.

### Insight and spatial non-insight problem solving: a general competence

Our findings unexpectedly revealed that solving spatial NIPs resembled solving IPs rather than verbal NIPs, since both benefited from similar cognitive contributors and endured the suppression of inner speech and the non-verbal attentional load. Twenty-one percent of the participants reported experiencing an impasse when solving spatial NIPs, whereas fewer than 2% experienced an impasse when solving verbal NIPs. These findings suggest that as the individual progresses through the process of solving spatial NIPs, they may arrive at problematic states, and consequently, should either switch or update the current sub-goal and procedure. When solving verbal NIPs, however, the individuals, who follow a known procedure, progress and feel that they know what to do. They are not stuck at an impasse and it is likely that failing to solve the problem results from insufficient time to complete the procedure or from an error made along the way.

NIPs are considered routine problems for which solvers retrieve an incremental solution procedure they already know; as in multiplying two-digit numbers (Dow & Mayer, 2004). Participants in this study solved verbal NIPs through a planned series of steps seemingly retrieved as known techniques, probably based on abundant experience with such problems widely practiced in educational settings (DeYoung et al., 2008). Conversely, spatial NIPs are less common in academic settings and not routinely practiced as verbal NIPs. Participants might not have solved these problems previously or had no stored, pre-prepared solving procedures in their memory. Hence, as in IPs, spatial NIPs can be considered as non-routine problems requiring unfamiliar solving methods.

This study showed that solving spatial NIPs does not depend on attention resources; reinforcing our argument that they are not solved by retrieving a multi-step procedure that needs to be retained in WM, but by creating new procedures or by trial and error. Lidstone et al. (2010) reached a similar conclusion when the Tower of Hanoi, a classic spatial NIP, showed no articulatory suppression effect on its performance. The researchers suspected that its solution did not rely on planning, so they conducted a second experiment in which participants had to plan the solution rather than concretely performing it. In the latter experiment, articulatory suppression was detrimental to performance. Hence, the standard Tower of Hanoi, which was also applied in the current study with other typical spatial NIPs, did not elicit advance planning.

Apparently, both spatial NIP and IP solving are neither speech-based nor attention demanding. They require generating new paths to solutions rather than retrieving known procedures. They significantly rely on convergent, near and spatial analogical skills, and flexible thinking. Seemingly, these cognitive skills underlie the ability to generate new procedures and/or solutions in general, regardless of the nature of the problem. One might conclude that insight might not be a special ability, but a general competence.

### Examining concurrent models of insight problem solving: a composite theory

The ostensible conflicting results of our research reflect the existing controversies in the literature. On the one hand, this study provides evidence that insight processing is unique, not attention demanding, and not speech reliant, as IP solving was consistently differentiated from verbal NIP solving and was affected neither by articulatory nor visuospatial sketchpad suppressions. These findings support the special-process theory. On the other hand, our findings consistently showed that spatial NIP solving resembles IP solving, thus suggesting that insight might not be that special. Both spatial NIP and IP solving significantly benefited from analogical and convergent thinking, and only to a lesser extent by flexible thinking, thus, indicating analytic processing, as posited by the business-as-usual theory.

In addition, approximately 60% of participants did not experience an impasse while trying to solve IPs silently, and only 8% of the problems reported as solved via insight evoked an impasse. Similarly, Fleck and Weisberg (2013) reported that 53% of their participants did not encounter an impasse, and 7% of insightful solutions were obtained following one. These findings appear to contradict the special-process theory, which conceptualizes an impasse as a crucial stage that typically gives rise to restructuring and, in turn, leads to the insightful solution. Moreover, impasses do not exclusively characterize IP solving as results of this study showed that processes involved in spatial NIP solving might also include a state of an impasse. Stuyck et al. (2021) reached similar conclusions because in their study the experience of impasse was associated with word puzzles solved with or without insight. Nonetheless, impasses are not accounted for by the business-as-usual theory.

Clearly, neither theory can fully explain the mechanism of insight, yet results of this study suggest that neither of them should be rejected. We propose that these theories are unequally complementary: the business-as-usual theory accounts for the major role in IP solving, similar to NIP solving, while the special-process theory plays a more minor role. Each theory relates to a different aspect of the insightful process as well a different way of reaching the insightful solution. The business-as-usual theory features the core typical analytic mode of solving that stresses the contributions of analogical and convergent skills as shown in this study. The special-process theory presents the milestones of insight: impasse and a sudden "Aha!" moment thus, addresses the unconventional insightful mode of thinking as only a minor portion of solutions included both experiences. Overall, the present findings are consistent with the business-as-usual theory, supporting IP and NIP solving as analytic in nature. Analytic thinking explained nearly half of the variance in IP solving, whereas divergent thinking explained only 2%. The associative, unconscious, special solution process, delineated by the special-process theory, is indeed unique. However, while proponents of this theory, claim it is unique since IPs alone are solved via this special process, the current results contradict their view. The special process can support NIP solving as well. Thus, it is special simply because it is rare.

Weisberg (2015) also addressed IP solving from the cognitive process perspective, suggesting an integrated outline of four-stage problem solving. In his model, the first three stages are analytic in nature. Each of them may produce a solution otherwise, either different solving methods are applied, or the representation of the problem is restructured due to the new information brought by the failure. This dynamic analytic process may recycle itself until it triggers an appropriate restructuring and an insightful solution. Only if the process exhausts itself, and the solver enters an impasse, should stage four occur evoking a solution through insight. Similarly, Chuderski and Jastrzębski (2018) proposed that only occasionally do the standard analytic problem-solving methods require additional insight processing. They described IP solving as "nothing special with special add-ons". In their study, special add-ons such as increased tendencies to decouple from ineffective approaches, and to restart the solving process, only marginally contributed to IP solving. The stage four in Weisberg's model, the Chuderski and Jastrzębski's special add-ons, and the fluency, flexibility, and originality factors (i.e., divergent thinking) in our study reflect less frequently used solution strategies whereas the analytic strategies are standard. Therefore, based on these studies, we may combine the special-process and the business-as-usual theories at the process level.

**Fig. 4:**
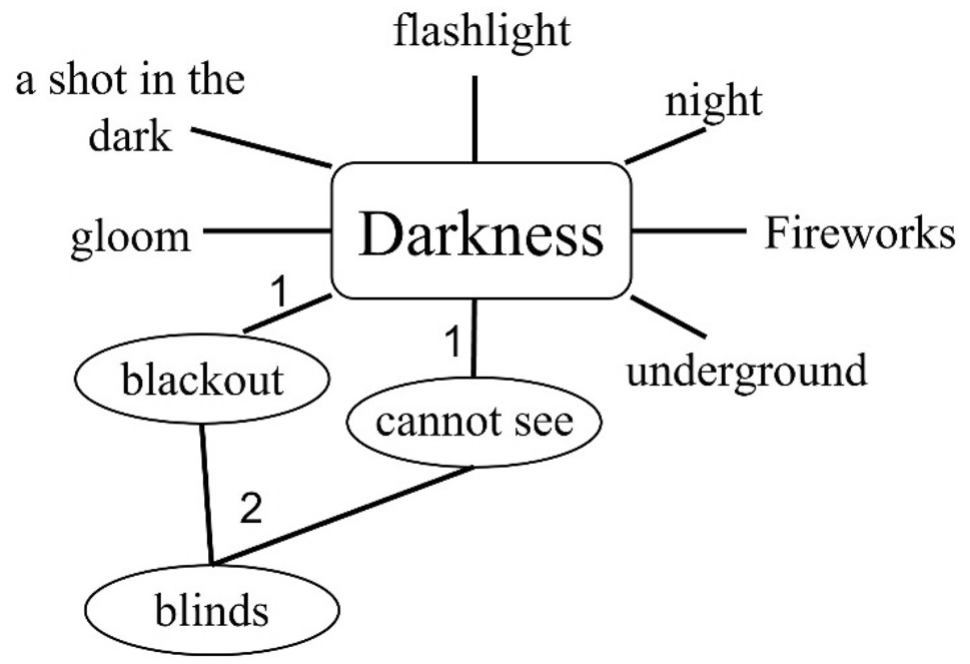
Plausible solution procedure that combines divergent (1) and convergent (2) thinking for the "blind" insight problem

The current study not only reinforces previous conclusions, but also gives rise to new findings. In contrast to verbal NIPs, solutions of IPs and spatial NIPs benefited from analogical thinking, as predicted by the business-as-usual theory, and benefited less from divergent thinking, which characterizes the special-process theory. Analogical thinking yields new assumptions about the problem and divergent thinking produces disparate ideas. Connecting these assumptions and ideas by a new association via convergent thinking might surprisingly lead to a solution. Figure 4 presents an example of an optional solution procedure that combines divergent and convergent thinking for the following IP used in this study: A man was reading a book when the lights went out. Although the room was completely dark, the man continued to read. How was that possible? Initially, varied associations could be triggered by "darkness" through divergent thinking, for example, **blackout**, **cannot see**, etc. Then a few could be linked together by a new association through convergent thinking, for example, "**blinds**", as window covers in times of blackouts, and as people with visual impairments. Once reaching the idea of "blinds", the problem is quickly solved—the man was reading Braille.

Results of this study suggest that the combined account explains not only solutions of IPs, but also solutions of spatial NIPs. Apparently, it accounts for the generation of new solutions rather than the retrieval of known solution procedures. Thus, insight can be considered a particular case of the former. The combined strategies support the generation of unfamiliar solutions to both problems that require lateral thinking and linear thinking. Whether the problems are classified as "insight problems" or not is irrelevant. We conclude that the definition of insight should be shifted towards focusing on the strategy rather than on the task. Task-related considerations should include whether the tasks require retrieving of existing solution procedures or creating new ones.

A new question may arise as to whether insight is rare for other than process-related factors? In our previous study, we attempted to answer this question by addressing IP solving from a person-centered perspective (Salmon-Mordekovich & Leikin, 2022). Results showed that experts on IP solving, constituting 12% of the sample, had unique characteristics related to creative and intellectual competencies. They demonstrated higher abilities on verbal divergent thinking and associative combination than successful IP solvers, who were somewhat less successful than the experts and than poor IP solvers. Not only did they retrieve creative ideas more fluently, but they also were better at combining ideas through novel association. Therefore, this small group of individuals was characterized as "highly associative and verbally creative". Moreover, while the successful solvers benefited from excellent WM capacity, the experts did not. Thus, we suggested that although experts and successful solvers have excellent analytic skills, the experts prefer to utilize insight strategy, while successful solvers, to a lesser extent, tend to apply analytic strategy. Thus, the special-process and business-as-usual theories may be combined at the individual level as well. Although each individual is endowed with both analytic and insight thinking, only a small group seems to favor or excel in the insight strategy.

The theoretical conflict in the literature might also stem from task-related differences among studies (e.g., modality, size of solution search space). According to this study, a study that compares solving IPs with spatial NIPs may find similarities between the two and thus would confirm the business-as-usual theory, whereas a study that contrasts solving IPs with verbal NIPs may find significant differences and therefore would support the special-process theory. Moreover, even two variants of the same problem may trigger different solution strategies, analytic or insight, due to size differences in the solution search space (Ash & Wiley, 2006). Additionally, demonstrating that the experimental conditions had no effects on IP solving can be viewed as support for automatic, unconscious processing, as the special-process theory posits. However, it could stem from a small initial solution search space that does not burden attentional capacity (Ash & Wiley, 2006) or from generating solutions by lateral thinking rather than retrieving multi-step solutions, as the results of this study suggest.

## Conclusions

From a theoretical perspective, this study reinforces an integrative approach: each theory relates to a different mode of thinking by which insightful solutions can be produced as well as to different features of the problem-solving process. To the best of our knowledge, no study has directly tested the role of analogical thinking as a person’s individual ability in IP solving. This study differentiated near from far verbal analogical thinking, a distinction that proved to be essential, and identified the near and spatial analogical abilities as substantial contributors to insight in particular, and to the creation of new solutions in general, including analytic ones. Analogous information is retrieved and processed in a multi-directional manner, as ideas produced by associative and flexible thinking. We believe that an insightful solution is the product of the convergence of these analogous inferences and ideas and not of spontaneous associations. We assert that both analogical and convergent thinking are key skills of insight.

Empirically, we provide novel evidence: IP solving consistently resembles spatial NIP but differs from verbal NIP solving. The similarities may substantiate the business-as-usual theory while the differences support the special-process theory. Thus, from a practical perspective, the results alert us to methodological susceptibility to non-meticulous problem selection. Distinguishing problems by modality is essential to avoid misleading findings. Furthermore, the traditional classification of problems into insight and non-insight seems irrelevant. The appropriate distinction should consider whether the problems require the retrieval of existing solution procedures or the creation of new ones.

Spatial NIPs, like IPs, do not involve linguistic processing and are more abstract than verbal NIPs. Solving these problems requires different thinking than solving verbal NIPs, since, in solving IPs and spatial NIPs the individual generates the solution procedure rather than retrieving it from long-term memory. Although the nature of these solutions is different; i.e., linear for spatial NIP processing while more lateral for IP, they both rely on similar cognitive skills. We suggest that nurturing both verbal and spatial analogical skills and convergent thinking in students might enhance the individual's ability to produce novel solutions regardless of the problem type. One should not teach someone how to be a good spatial NIP solver or to be insightful, but rather should instill cognitive foundations that enable an individual to become one.

## Appendix A

Sample Verbal and Spatial Insight Problems

**Checkers.** Two men played five games of checkers and each won an even number of games, with no ties or forfeits. How is that possible?

(Solution: They were not playing against each other.)

**Four dots.** Connect all 4 dots with two straight lines without lifting your pencil from the paper.

Sample Verbal and Spatial Non-Insight Problems

**Committee.** Smith is a butcher and president of the street storekeepers’ committee, which also includes the grocer, the baker, and the pharmacist. They sit around a table.

- Smith sits on Jones’ left.
- Davis sits at the grocer’s right.
- Bailey, who faces Jones, is not the baker.

Assign each storekeeper to the correct store.

(Solution: Smith-butcher, Davis-baker, Bailey-pharmacist, Jones-grocer)

**Trace.** Without lifting your pencil from the paper, trace the figure below. No line cannot be traced more than once.

(One of the extreme points on either side must be the starting point.)
